# Influenza vaccination in Western Australian children: Exploring the health benefits and cost savings of increased vaccine coverage in children

**DOI:** 10.1016/j.jvacx.2023.100399

**Published:** 2023-10-18

**Authors:** Christopher C. Blyth, Parveen Fathima, Rebecca Pavlos, Peter Jacoby, Olivia Pavy, Elizabeth Geelhoed, Peter C Richmond, Paul V. Effler, Hannah C. Moore

**Affiliations:** a Wesfarmers Centre of Vaccines and Infectious Diseases, Telethon Kids Institute, University of Western Australia, Perth, WA, Australia; b School of Medicine, University of Western Australia, Perth, WA, Australia; c Department of Infectious Diseases, Perth Children’s Hospital, Perth, WA, Australia; d Department of Microbiology, PathWest Laboratory Medicine, QEII Medical Centre, Perth, WA, Australia; e School of Public Health, University of Sydney, Sydney, New South Wales, Australia; f Telethon Kids Institute, Perth Children’s Hospital, Perth, WA, Australia; g Department of Immunology, Perth Children’s Hospital, Perth, WA, Australia; h Department of General Paediatrics, Perth Children’s Hospital, Perth, WA, Australia; i Communicable Disease Control Directorate, Department of Health, Perth, WA, Australia; j School of Population Health, Curtin University, Perth, Western Australia, Australia

**Keywords:** Influenza, Influenza vaccination, Costs, Child

## Abstract

**Introduction:**

To assess potential benefits and direct healthcare cost savings with expansion of an existing childhood influenza immunisation program, we developed a dynamic transmission model for the state of Western Australia, evaluating increasing coverage in children < 5 years and routinely immunising school-aged children.

**Methods:**

A deterministic compartmental Susceptible-Exposed-Infectious-Recovered age-stratified transmission model was developed and calibrated using laboratory-notification and hospitalisation data. Base case vaccine coverage estimates were derived from 2019 data and tested under moderate, low and high vaccine effectiveness settings. The impact of increased coverage on the burden of influenza, influenza-associated presentations and net costs were assessed using the transmission model and estimated health utilisation costs.

**Results:**

Under base case vaccine coverage and moderate vaccine effectiveness settings, 225,460 influenza cases are expected annually across all ages. Direct healthcare costs of influenza were estimated to be A$27,608,286 per annum, dominated by hospital costs. Net cost savings of >$A1.5 million dollars were observed for every 10 % increase in vaccine coverage in children < 5 years. Additional benefits were observed by including primary school age children (5–11 years) in the funded influenza vaccination program - a reduction in cases, presentations, hospitalisations and approximately $A4 million net costs savings were observed for every 10 % increase in coverage. The further addition of older children (12–17 years) resulted in only moderate additional net cost savings figures, compared with a 5–11year-old program alone. Net costs savings were predominantly derived by a reduction in influenza-associated hospitalisation in adults.

**Conclusions:**

Any increase in influenza vaccine coverage in children < 5 years, above a base case of 50 % coverage resulted in a substantive reduction in influenza cases, presentations, hospitalisations and net costs when applied to the West Australian population. However, the most impactful pediatric program, from both a disease prevention and costs perspective, would be one that increased vaccination coverage among primary-school aged children.

## Highlights

• Using a deterministic compartmental age-stratified transmission model developed to simulate the West Australian population, increased vaccine coverage in children younger than 5 years was associated with a significant reduction in influenza morbidity and net costs in all age groups.
• Increased vaccine coverage in primary school age children (5–11 years) was a highly effective strategy resulting in an even greater reduction in influenza morbidity and net costs (in all age groups), compared with increased coverage in preschool children alone.
• The relative impact of increased vaccine coverage in children aged 5–17 years was moderate when compared with the benefits of programs focusing on children 5–11 years alone, with only moderate additional reductions in morbidity and net costs.

## 1 Introduction

Immunisation is one of the most cost-effective life-saving interventions in public health [1]. Influenza remains a frequently diagnosed vaccine preventable disease [2] yet surveillance data underestimate the likely burden of influenza in the community. Influenza is a common cause for paediatric hospitalisation [3], [4], [5], [6], and the most common vaccine-preventable cause for paediatric death [7]. Children less than five years of age are at greatest risk of hospitalisation [8].

Influenza immunisation is recommended by the Australian Technical Advisory Group on Immunisation for all Australians aged ≥ 6 months [9] and funded for those at greatest risk of severe disease. Nationally-funded programs were initially established under the National Immunisation Program (NIP) for children ≥ 6 months of age with comorbidities predisposing them to severe outcomes following influenza infection. Additional programs were established for Aboriginal and/or Torres Strait Islander (hereafter referred to as First-Nations) children aged less than 5 years [10]. State-funded whole-of-population programs for all children aged 6 months to < 5 years were established in Western Australia (WA) in 2008 and other Australian jurisdictions in 2018 [10]. Informed by an independent assessment of the benefits, costs and cost-effectiveness of an expanded influenza immunisation program undertaken by the Pharmaceutic Benefits Advisory Committee (Australian Government), influenza immunisation was funded under the NIP for all Australian children aged 6 months to < 5 years in 2020. A further funded WA state-led program has provided influenza immunisation for all primary school aged children (5-11 years) across WA since 2020.

Studies have demonstrated the direct effectiveness of influenza vaccines against influenza infection in immunised children [3], [4], [11]. Ecological studies [12], [13] and more recently, clinical trials [14], [15], have also demonstrated the significant indirect effects of childhood influenza immunisation on unimmunised children and adults (i.e. indirect protection is provided to the community [the herd] by immunising children). Loeb *et al*. demonstrated a 61 % reduction in influenza infections in unimmunised children and adults in Hutterite communities where children aged 3–15 years were immunised against influenza compared with communities where children received placebo immunisation [14]. Both direct and indirect effects of immunisation have been critical to analyses demonstrating that these programs are cost-effective. [16], [17], [18].

We sought to assess the potential benefits and net direct healthcare cost savings associated with increased vaccine coverage and further expansion of the current < 5 years old childhood influenza immunisation program. Our specific aims were to: i) develop a dynamic influenza transmission model to replicate influenza epidemiology in the population of Western Australia under varying influenza immunisation program settings; ii) evaluate potential net direct healthcare cost savings of the WA preschool (<5 years of age) influenza immunisation program utilising the dynamic model and iii) explore potential health and economic benefits associated with increasing vaccine coverage in preschool children and expanded programs immunising school-aged children.

## 2 Methods

### 2.1 Context

A deterministic compartmental Susceptible-Exposed-Infectious-Recovered (SEIR) age-stratified transmission model was developed to estimate the burden of influenza infections, and influenza-associated hospitalisations in WA under different vaccination scenarios. These data were then used to estimate, the direct healthcare costs and net cost savings of varying programs. The model was developed for the state of Western Australia, encompassing a third of the Australian land mass, with a population of 2.7 million people [19], and calibrated using hospitalisation data provided by WA Health. In WA, nearly 80 % of the population live within the metropolitan area and 3.3 % identify as being of First Nations origin. The climate ranges from the temperate south, traditionally with winter influenza outbreaks to the tropical north with year-round influenza activity.

### 2.2 Model structure

The SEIR transmission model was stratified by the following age groups: <5 years, 5–11, 12–17, 18–44, 45–64 and ≥ 65 years, based on the published SEIR transmission model from the R package *fluEvidenceSynthesis* (see epidemiological model supplement) [20]. The epidemiological model computes the weekly number of influenza infected individuals in each age group over a year on the basis of number of contacts, probability of disease transmission, incubation period, recovery rate and immunity, either following natural infection or induced by immunisation. The model allows for different vaccine effectiveness and coverage values for each age group and variable timing of immunisation. We used contact data (by age-groups) from the UK arm of the POLYMOD study [21] supplied with *fluEvidenceSynthesis* to describe the pattern of contacts between individuals in different age groups. We also implemented an extension to the supplied model by incorporating seasonal forcing in the transmissivity (i.e. the probability of a contact between an infected individual and a susceptible individual resulting in infection) parameter to correspond with observed winter peaks of influenza activity experienced in the temperate climate of southern WA where the vast majority of West Australians reside.

### 2.3 Calibration of the epidemiological model to WA data

The model was first calibrated by fitting model parameters to the observed hospitalisation data (2014–2016) using least squares optimisation. Weekly influenza-coded hospital admissions for these years were used to ensure optimal fit of the following model parameters: base transmissivity, seasonal amplitude of transmissivity variation, timing of peak transmissivity, incubation period, infectiousness period, immune fraction at the start of each year, number infected at the start of each year, and hospitalised fraction of total infections (values for < 5, 5–17, 18–64 and ≥ 65 years age groups). WA 2019 demographic data of population counts by year of age, obtained from the Australian Bureau of Statistics [19], were used to define the structure of the population in terms of age in the model.

### 2.4 Estimating the impact of influenza infection and the immunisation program

The epidemiological model predicted the number of influenza infections in a year in WA under varying vaccination scenarios. For final predictions, a total number of influenza cases was derived from the average case count under each of the 2014 to 2016 model parameters. The highest and lowest number of cases under these varying parameters across the three years was used to determine the range.

Influenza-associated hospitalisations were calculated by multiplying the number of total influenza cases (and range) by the age-specific influenza-related hospitalisation rate. ICU figures were determined by multiplying hospitalisation figures by age-specific ICU-admission rates, obtained from an ongoing Australian influenza surveillance program [4], [22]. By multiplying ICU figures by age-specific length-of-stay (LoS) data and subtracting these from the mean age-specific hospital LoS, non-ICU bed days were determined (see data supplement).

Emergency department (ED) presentations were calculated by dividing the model-estimated influenza-associated hospitalisation numbers (and range) [23], [24] by the known proportion of ED presentations requiring admission. As total ED presentations included both admitted and non-admitted numbers, subtracting admissions from the total ED presentations allowed us to determine the number of non-admitted ED presentations.

General Practice (GP) visits were calculated by applying previously published age-specific proportions of influenza-like illness episodes seeking primary care review by the total influenza infections (and range) estimated by the model. Published estimates utilised varied by age from 37 % (18-49 years) to 67 % (0–4 years) [25] approximating estimates observed in Australian adults in non-pandemic years [26].

### 2.5 Analysis of healthcare costs

The direct healthcare costs of influenza infection, estimated in Australia dollars (A$) included influenza-associated GP visits, ED presentations, influenza-associated hospitalisations (including non-ICU admissions and non-ICU bed days) and ICU admissions (incorporating ICU bed days). Healthcare cost data.

(2018–2019; see data supplement) were referenced from the latest available data sources [23], [27], [28]. Influenza-related GP visits were multiplied by A$38.75, the cost of a GP appointment, giving us a total costs for influenza-related GP appointments. Influenza-associated ED presentations were estimated from national hospitalisation data. Admitted and non-admitted presentation numbers were multiplied by A$1,464 and A$675 respectively, giving us the total cost of influenza-related ED presentations. Using published estimates, the costs of an influenza-related non-ICU stay was A$5,864, with a mean length-of-stay (LoS) of 2.2 days (i.e. estimated to be A$2,665 per non-ICU day). Total age-specific ICU bed-days were multiplied by A$4,375 with the combined total cost giving us the total of influenza-related hospitalisation.

The cost of delivering the vaccine program considered the actual vaccine cost (personal communication, Dr Paul Effler, WA Health) and the current cost of a GP consultation [27]. Sensitivity analyses were conducted to simulate less expensive models of vaccine delivery (e.g. mass immunisation clinic; school-based immunisation program), by halving the immunisation delivery costs.

### 2.6 The base case

The base case for vaccine coverage (VC) in age group was derived from 2019 data provided by WA health (50 % coverage in those < 5 years, 5–11 years: 10 %, 12–17 years: 10 %, 18–44 years: 20 %, 45–64 years: 20 %, ≥65 years: 75 %). To account for difference in vaccine effectiveness, the model was run under moderate vaccine effectiveness settings (VE = 60 % in those aged < 65; 40 % in those age ≥ 65 years) with sensitivity analyses undertaken under low VE (-20 % overall: ie VE = 40 % in those aged < 65; 20 % in those age ≥ 65 years) and high VE settings (+20 % overall: i.e. VE = 80 % in those aged < 65; 60 % in those age ≥ 65 years).

To explore the impact of variation to the immunisation program on the total burden of influenza and direct healthcare costs, three scenarios were developed. Scenario one varied VC in children < 5 years (base case = 50 %, VC ranging from 20 to 80 %) in moderate VE settings with sensitivity analyses undertaken in low (-20 %) and high (+20 %) VE settings. Scenario two varied VC in children 5–11 years (base case 10 %, VC ranging from 10 to 80 % whilst maintaining base case settings in all other age groups including children < 5 years) and scenario three varied VC in children 5–17 years (base case 10 %, VC ranging from 10 to 80 % whilst maintaining base case settings for other groups). Sensitivity analyses incorporating low and high VE settings were then applied to scenarios two and three.

## 3 Results

Our dynamic transmission model generated the annual influenza infection and health morbidity indicators for each age group. Under base-case vaccine coverage and moderate vaccine effectiveness settings, an average total of 225,460 influenza cases were predicted (with an estimated range of 142,065 to 267,549 episodes). These resulted in 103,772 GP presentations (65,518 to 123,111), 4,187 ED presentations (1865 to 6758), 1002 hospitalisations (485 to 1532) not requiring ICU admission and 133 hospitalisations (61 to 210) requiring ICU admission (supplemental table 2). When compared with the total number of estimated cases, a greater proportion of those < 5 years and ≥ 65 years required hospitalisation (<5 years: 0.73 % of influenza cases hospitalised; ≥65 years: 3.12 % of influenza cases hospitalised; 5–64 years: 0.33 % of influenza cases hospitalised). Vaccine effectiveness had a significant impact on the total influenza cases estimated (low vaccine effectiveness: 364,089 cases; high vaccine effectiveness: 128,765) as well as GP visits, ED presentations and hospitalisations.

### 3.1 Scenario 1: Adjusting vaccine coverage in children < 5 years

**Fig. 1:**
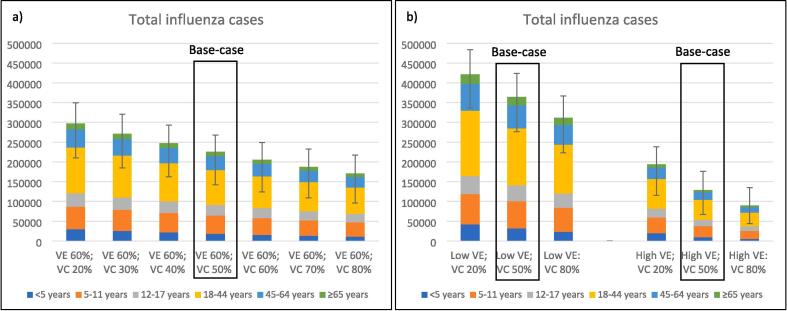
Overall influenza cases and range with varying vaccine coverage (VC) in children < 5 years under moderate vaccine effectiveness (VE) settings (a) with sensitivity analyses using low and high VE settings (b) (scenario 1).

Varying VC in children < 5 years (VC: 20 % to 80 %; base case 50 %) significantly impacted on total influenza cases (Fig. 1a-b) and influenza-associated GP visits, ED presentations and hospitalisations (supplemental Fig. 1a-d) in all vaccine effectiveness settings (supplementary table 2). The impact was observed in all age groups including those > 5 years of age.

**Fig. 2:**
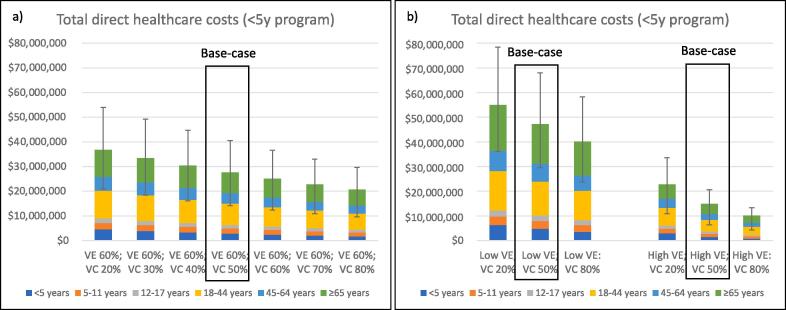
Overall direct healthcare costs and range with varying vaccine coverage in children < 5 years under moderate vaccine effectiveness (VE) settings (a) with sensitivity analyses using low and high VE settings (b) (scenario 1), All figures in Australian dollars.

The overall direct healthcare costs (Fig. 2a-b) and costs of influenza-associated GP presentations, ED presentations and hospitalisations (supplemental Fig. 2a-d) are similarly presented demonstrating the impact of varying VC in children < 5 years in similar VE settings (supplementary table 3). Using the base-case vaccine coverage and moderate vaccine effectiveness settings, the annual cost to the health care system for influenza-associated GP visits, ED presentations and hospitalisations was estimated to be A$27,608,286 (range: A$14,141,938 to A$40,541,389; Table 1). The predominant driver of overall direct health care costs was hospitalisation (non-ICU hospital costs: A$16,232,038 [58.8 %]; ICU hospital costs: A$3,663,705 [13.2 %]; ED presentation cost: A$3,721,390 [13.4 %] and GP visit costs: A$4,021,153 [14.6 %]).

**Table 1:** Net cost savings of an increase in influenza vaccine coverage in children < 5 years moderate vaccine effectiveness (VE) settings with sensitivity analyses using low and high VE settings (scenario 1).

	Vaccine Uptake in children (<5years)	Direct health care costs	Cost Reduction (from base case scenario)	Vaccination cost	Net Cost Savings (relative to base case)
Primary analysis	Moderate vaccine effectiveness [60 % (6 m-64y), 40 % (65 + )] Vaccine uptake [<5 years: 20–80 %, 5–17 years: 10 %, 18–64 years: 20 %, ≥65 years: 75 %]				
	20 %	$36,721,573 ($20,954,867 to $53,988,318)	-$9,113,287 (-$13,466,928 to -$6,812,928)	-$2,523,573	-$6,589,714 (-$10,923,356 to -$4,289,356)
	30 %	$33,448,353 ($18,422,969 to $49,241,959)	-$5,840,067 (-$8,700,570 to -$4,281,030)	-$1,682,382	-$4,157,686 (-$7,018,188 to -$2,598,648)
	40 %	$30,402,938 ($16,140,143 to $44,740,076)	-$2,794,651 (-$4,198,686 to -$1,998,204)	-$841,191	-$1,953,461 (-$3,357,496 to -$1,157,013)
	50 % (base case)	$27,608,286 ($14,141,938 to $40,541,389)			
	60 %	$25,043,584 ($12,391,619 to $36,590,868)	$2,564,701 ($1,750,319 to $3,950,521	$841,191	$1,723,510 ($909,129 to $3,109,330)
	70 %	$22,719,583 ($10,874,678 to $32,989,346)	$4,888,703 ($3,267,260 to $7,552,043)	$1,682,382	$3,206,321 ($1,584,879 to $5,869,661)
	80 %	$20,616,394 ($9,562,517 to $29,653,262)	$6,991,892 ($4,579,421 to $10,888,128)	$2,523,573	$4,468,319 ($2,055,848 to $8,364,555)
Sensitivity analyses	Low VE [VE estimates −20 % lower in all age groups]				
	20 %	$54,917,020 ($36,087,102 to $78,437,861)	-$7,719,804 (-$10,414,920 to -$6,556,993)	-$2,523,573	-$5,196,231 (-$7,891,348 to -$4,033,420)
	50 % (base case)	$47,197,216 ($29,530,109 to $68,022,941)			
	80 %	$40,167,667 ($23,756,736 to $58,257,967)	$7,029,549 ($5,772,373 to $9,764,973)	$2,523,573	$4,505,976 ($3,249,800 to $7,241,400)
	High VE [VE estimates + 20 higher in all age groups]				
	20 %	$22,767,433 ($10,835,993 to $33,565,447)	-$7,921,976 (-$12,939,367 to -$4,453,791)	-$2,523,573	-$5,398,403 ($-10,415,794 to -$1,930,219)
	50 % (base case)	$14,845,457 ($6,382,201 to $20,626,080)			
	80 %	$10,126,953 ($4,299,391 to $13,301,375)	$4,718,504 ($2,082,810 to $7,845,987)	$2,523,573	$2,194,931 (-$440,762 to $5,322,414)

VE = Vaccine effectiveness, All figures in Australian dollars.

Any increase in VC in children < 5 years above the base case was associated with significant net direct healthcare costs savings due to a reduction in influenza cases in all age groups and associated healthcare costs (Table 1). An incremental increase in VC by 10 %, 20 % and 30 % above the base case (VC of 50 %) was associated with an increase in net savings of A$1,723,510 (A$909,129 to A$3,109,330), A$,3,206,321 (A$1,584,879 to A$5,869,661) and A$4,468,319 (A$2,055,848 to A$8,364,555) respectively. A reduction of VC was associated with an increased direct costs and negative net cost savings. These observations were explored, through sensitivity analyses, with consistent trends in low and high VE settings.

### 3.2 Scenario 2 & 3: Adjusting vaccine coverage in children 5–11 years and 5–17 years

**Fig. 3:**
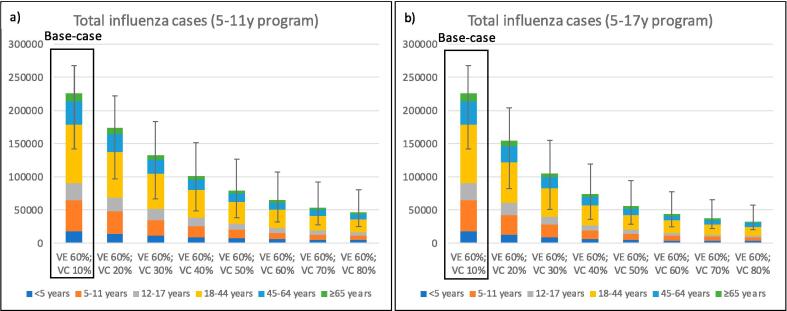
Overall influenza cases (and range) with varying vaccine coverage in children 5–11 years (scenario 2) and 5–17 years (scenario 3) under moderate vaccine effectiveness (VE) settings (a, b) Uptake in children < 5 years are maintained at base-case settings (50 %).

**Fig. 4:**
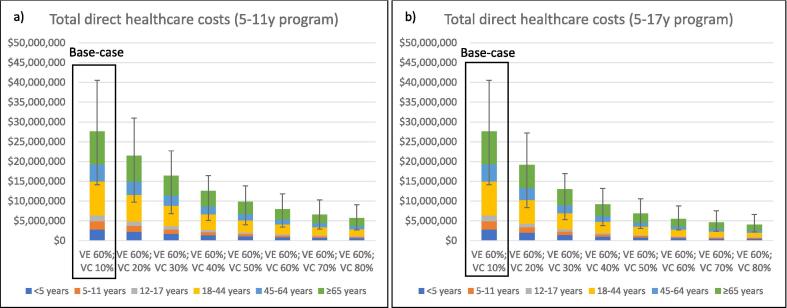
Overall direct healthcare costs (and range) with varying vaccine coverage in children 5–11 years (scenario 2) and 5–17 years (scenario 3) under moderate vaccine effectiveness (VE) settings (a, b) Uptake in children < 5 years are maintained at base-case settings (50 %). All figures in Australian dollars.

The potential benefit of increased vaccine coverage in children 5–11 years (scenario 2) and 5–17 years (scenario 3) were explored utilising the model and assessing total cases, influenza-associated GP presentations, ED presentations and hospitalisations and direct healthcare costs (Fig. 3, Fig. 4; Supplemental Fig. 3, Fig. 4). An increase in coverage from the base case (50 % in < 5 years; 10 % in children 5–17 years) to 50 % coverage in children 5–11 years (Scenario 2) and 5–17 years (Scenario 3) resulted in a 64.7 % and 75.4 % reduction in total cases respectively, a 64.4 % and 75.1 % reduction in influenza-associated hospitalisations and a 64.4 % and 75.1 % reduction total costs (Fig. 3, Fig. 4). A reduction in cases and costs was observed in all age groups including in those aged ≥ 65 years (62.2 % and 72.5 % reduction in total cases and total costs).

Increasing VC in children ≥ 5 years from base case was associated with significant net savings, greater than were observed with increased coverage in children < 5 years (Table 2, Table 3). An incremental increase in VC by 10 %, 20 % and 30 % above baseline (VC of 10 %) was associated with an increase in net saving of A$4,991,890, A$8,847,563 and A$11,491,757 in Scenario 2. This was compared with net savings of A$6,381,059, A$10,406,119 and A$12,169,577 in Scenario 3, demonstrating that the incremental net cost saving of an adolescent program provided only a moderate benefits over a 5–11 only program. Of note, the net cost savings plateaued and (were lost in high VE settings) with VC increases above 50 % in children 5–11 years and 40 % in children 5–17 years suggesting that likely threshold where any further increases were of limited additional economic benefit.

**Table 2:** Estimated Net Cost Savings (and range) of an increase in influenza vaccine coverage in children 5–11 years under moderate vaccine effectiveness (VE) settings with sensitivity analyses using low and high VE settings (scenario 2).

	Vaccine Uptake in children (<5years)	Direct health care costs	Cost Reduction (from base case scenario)	Vaccination cost	Net Cost Savings (relative to base case)
Primary analysis	Moderate vaccine effectiveness [60 % (6 m-64y), 40 % (65 + )] Vaccine uptake [<5 years: 20–80 %, 5–17 years: 10 %, 18–64 years: 20 %, ≥65 years: 75 %]				
	10 % (base case)	$27,608,286 ($14,141,938 to $40,541,389)			
	20 %	$21,438,894 ($9,741,980 to $31,001,738)	$6,169,392 ($4,399,958 to $9,539,651)	$1,177,502	$4,991,890 ($3,222,455 to $8,362,148)
	30 %	$16,405,717 ($6,851,558 to $22,693,429)	$11,202,568 ($7,290,380 to $17,847,960)	$2,355,055	$8,847,563 ($4,935,375 to $15,492,955)
	40 %	$12,584,021 ($5,084,331 to $16,197,432)	$15,024,264 ($9,057,607 to $24,343,957)	$3,532,507	$11,491,757 ($5,525,099 to $20,811,449)
	50 %	$9,836,519 ($4,026,587 to $11,618,279)	$17,771,767 ($10,115,351 to $28,923,110)	$4,710,010	$13,061,757 ($5,405,341 to $24,213,100)
	60 %	$7,938,030 ($3,400,952 to $8,570,622)	$19,670,256 ($10,740,986 to $31,970,767)	$5,887,513	$13,782,743 ($4,853,473 to $26,083,254)
	70 %	$6,609,263 ($2,947,329 to $6,561,447)	$20,999,022 ($11,194,609 to $33,979,942)	$7,065,015	$13,934,007 ($4,129,593 to $26,914,927)
	80 %	$5,684,794 ($2,708,606 to $5,282,090)	$21,923,491 ($11,433,332 to $35,259,299)	$8,242,518	$13,680,973 ($3,190,814 to $27,016,781)
Sensitivity analyses	Low VE [VE estimates −20 % lower in all age groups]				
	10 % (base case)	$47,197,216 ($29,530,109 to $68,022,941)			
	30 %	$36,815,604 ($20,247,941 to $53,965,220)	$10,381,612 ($9,282,167 to $14,057,720)	$2,355,055	$8,026,607 ($6,927,162 to $11,702,716)
	50 %	$27,379,553 ($12,910,032 to $39,983,989)	$19,817,662 ($16,620,077 to $28,038,951)	$4,710,010	$15,107,652 ($11,910,067 to $23,328,941)
	70 %	$19,752,026 ($8,194,464 to $27,685,944)	$27,445,190 ($21,335,645 to $40,336,996)	$7,065,015	$20,380,175($14,270,629 to $33,271,981 )
	High VE [VE estimates + 20 higher in all age groups]				
	10 % (base case)	$14,845,457 ($6,382,201 to $20,626,080)			
	30 %	$7,395,930 ($3,245,688 to $8,103,216)	$7,449,520 ($3,136,513 to $12,522,863)	$2,355,055	$5,094,517 ($781,508 to $10,167,858)
	50 %	$4,746,033 ($2,458,163 to $4,256,403)	$10,099,424 ($3,924,037 to $16,369,676)	$4,710,010	$5,389,414 (-$785,973 to $11,659,666)
	70 %	$3,665,498 ($2,100,334 to $3,003,257)	$11,179,958 ($4,281,866 to $17,622,823)	$7,065,015	$4,114,943 (-$2,783,149 to $10,557,807)

VE = Vaccine effectiveness, All figures in Australian dollars.

**Table 3:** Estimated Net Cost Savings (and range) of an increase in influenza vaccine coverage in children 5–17 years under moderate vaccine effectiveness (VE) settings with sensitivity analyses using low and high VE settings (scenario 3).

	Vaccine Uptake in children (<5years)	Direct health care costs	Cost Reduction (from base case scenario)	Vaccination cost	Net Cost Savings (relative to base case)
Primary analysis	Moderate vaccine effectiveness [60 % (6 m-64y), 40 % (65 + )] Vaccine uptake [<5 years: 20–80 %, 5–17 years: 10 %, 18–64 years: 20 %, ≥65 years: 75 %]				
	10 % (base case)	$27,608,286 ($14,141,938 to $40,541,389)			
	20 %	$19,129,222 ($8,352,415 to $27,232,711)	$8,479,064 ($5,789,524 to $13,308,678)	$1,177,502	$6,381,059 ($3,691,519 to $11,210,673)
	30 %	$13,006,156 ($5,309,464 to $16,944,154)	$14,602,129 ($8,832,474 to $23,597,235)	$2,355,055	$10,406,119 ($4,636,464 to $19,401,225)
	40 %	$9,144,694 ($3,770,510 to $13,154,558)	$18,463,591 ($10,371,429 to $27,386,831)	$3,532,507	$12,169,577 ($4,077,414 to $23,854,324)
	50 %	$6,874,080 ($3,062,087 to $10,597,425)	$20,734,205 ($11,079,852 to $33,578,661)	$4,710,010	$12,342,186 ($2,687,832 to $25,186,641)
	60 %	$5,487,729 ($2,675,919 to $8,769,080)	$22,120,557 ($11,466,019 to $31,772,309)	$5,887,513	$11,630,532 ($975,994 to $25,884,796)
	70 %	$4,616,818 ($2,376,202 to $7,516,792)	$22,991,468 ($11,765,736 to $33,024,597)	$7,065,015	$10,403,438 (-$822,294 to $25,959,582)
	80 %	$4,055,885 ($2,222,275 to $6,628,626)	$23,552,401 ($11,919,663 to $33,912,763)	$8,242,518	$8,866,366 (-$2,766,372 to $25,670,245)
Sensitivity analyses	Low VE [VE estimates −20 % lower in all age groups]				
	10 % (base case)	$47,197,216 ($29,530,109 to $68,022,941)			
	30 %	$32,483,269 ($16,759,722 to $47,686,154)	$14,713,947 ($12,770,387 to $20,336,786)	$4,196,010	$10,517,938 ($8,574,377 to $16,140,777)
	50 %	$20,508,596 ($8,672,306 to $28,929,188)	$26,688,620 ($20,857,803 to $39,093,752)	$8,392,020	$18,296,600 ($12,465,783 to $30,701,733)
	70 %	$12,747,667 ($5,033,690 to $15,931,250)	$34,449,549 ($24,496,419 to $52,091,690)	$12,588,030	$21,861,519 ($11,908,389 to $39,503,660)
	High VE [VE estimates + 20 higher in all age groups]				
	10 % (base case)	$14,845,457 ($6,382,201 to $20,626,080)			
	30 %	$5,899,551 ($2,774,941 to $9,066,826	$6,014,919 ($3,607,260 to $11,559,254)	$4,196,010	$3,916,914 (-$588,750 to $7,363,244)
	50 %	$3,734,839 ($2,147,247 to $5,961,688)	$10,330,705 ($4,234,954 to $14,664,392)	$8,392,020	$4,036,690 (-$4,157,066 to $6,272,372)
	70 %	$3,000,124 ($1,937,380 to $4,287,881)	$11,571,118 ($4,444,821 to $16,338,199)	$12,588,030	$1,081,094 (-$8,143,209 to $3,750,169)

VE = Vaccine effectiveness, All figures in Australian dollars.

A further reduction in vaccine delivery costs, such as through a school-based program saw additional costs saving. Compared with a primary care-based program (Table 2, Table 3), an incremental increase in VC by 10 %, 20 % and 30 % above baseline (VC of 10 %) was associated with an increase in net saving of A$5,459,871, A$9,783,527 and A$12,895,702 in Scenario 2. This was compared with net saving A$7,769,543, $13,183,088 and $16,335,029 respectively in Scenario 3.

## 4 Discussion

Utilising a deterministic compartmental SEIR transmission model, calibrated on data for the state of Western Australia, current direct healthcare costs and estimated vaccine delivery costs, our results demonstrate that even modest improvements (e.g. a 10 % increase) in influenza vaccine coverage in children < 5 years beyond a base case of 50 % is likely to result in net cost savings. Further economic benefit is likely to be observed by including all primary school age children (5-11 years) in the funded influenza vaccination program. The addition of high school children (12-17 years) resulted in smaller net cost savings, suggesting only moderate additional economic benefit by extending a program into older children. A reduction in vaccine delivery costs, such as through school-based delivery, saw additional costs averted. These net costs savings were largely derived by a reduction in influenza-associated hospitalisation in adults, including the elderly, suggesting significant indirect benefits of the program.

The benefits of influenza vaccination are dependent on both vaccine effectiveness and achieving adequate vaccine coverage. Following a large influenza season in 2017 [4], state-based programs funding vaccine for children < 5 years were established in all Australian states [10]. Influenza vaccination coverage in Australia peaked in 2019 with 41.8 % of Australian children < 5 years receiving an influenza vaccination (52.0 % in WA) [29]. Lower coverage has been observed since 2020 (6 months to 5 years − 2020: 32.4 %, 2021: 24.5 % and 2022: 32.4 % [30], likely influenced by the near absence of influenza in Australia over the 2020–2021 period [31].

Influenza vaccination is not currently funded on the National Immunisation Program, for healthy non-Indigenous school aged children (ie 5 years and over) in Australia. There is a wealth of international data, using a variety of approaches, demonstrating the significant indirect effects of influenza vaccination [12], [13], [14], [15]), particularly when provided to school age children. These indirect effects are particularly observed in this age group as they considered major transmitters of influenza in communities, driven by numerous factors including susceptibility, viral shedding, behaviour and mobility. In addition to studies demonstrating the impact of vaccinating school age children on the total community burden of influenza, there are increasing international data demonstrating the economic benefits of such strategies [32], [33], [34], [35]. Similar to other investigators, our findings confirm that targeting primary school age children in Australia appears to result in the greatest economic benefit. Although there are a lack of published Australian data demonstrating these findings, this observation mirror previous independent modelling undertaken for the Commonwealth Department of Health (2016–2019: Hannah Moore; pers communication). Informed by these data, the WA state government has provided funded influenza immunisation to children 5 to 11 years since 2020. This has not been adopted in other Australian jurisdictions. Given similarities in population structure, climate, influenza epidemiology and dynamics, and access to health and vaccination services in Western Australia and other jurisdictions (both in Australia and similar southern hemisphere jurisdictions), these data suggest that a primary school age program is likely to be economically beneficial in non-West Australian settings and should be immediately considered. Data demonstrating both the direct and indirect benefits and cost-effectiveness of influenza must also be incorporated into public health campaigns to promote vaccine use in children.

This is the first contemporary Australian study to assess the economic benefits of modifications to the current influenza vaccination program, developed using real-world data and published health-care costs. The study has some limitations. The model did not specifically include Indigenous Australian (approximately 4 % of the WA population) and those with medical risk factors for severe influenza vaccination. This is relevant given they are already recommended and can access funded immunisation from 6 months of age. As no appropriate contact matrices were available at the time of study design, the study used contact matrices from the United Kingdom which may be different to those observed in Australia. Given that the vast majority of the population live in the temperate metropolitan area and Southwest region of Western Australia, the study assumed similar transmission parameters state-wide. The study assumed base case vaccine coverage using estimates published prior to 2020, yet more recent estimates suggest lower uptake, particularly in children < 5 years. With estimated cases, the study included direct health-care costs only, and did not consider other societal costs including absenteeism. It is possible, that by using the rebated GP cost, we underestimated the true costs of the vaccine program, although note that this is the predominant way to access influenza vaccination in Western Australia.

## 5 Conclusion

The results show that strengthening the current pre-school influenza vaccination program, through increased coverage in children < 5 years, and expansion to provide funded immunisation to primary school age children is expected to substantially reduce the burden of influenza disease and is likely to result in significant net cost savings to the community.

## Declaration of Competing Interest

The authors declare that they have no known competing financial interests or personal relationships that could have appeared to influence the work reported in this paper.

## Data Availability

Data will be made available on request.
