# Supplementary material for: Influenza vaccination in Western Australian children: Exploring the health benefits and cost savings of increased vaccine coverage in children
Source: Vaccine X. 2023 Oct 18;15:100399. doi: 10.1016/j.jvacx.2023.100399 (PMC10613898; doi:10.1016/j.jvacx.2023.100399)
Supplement: Supplementary data 1 [file mmc1.docx]

**model supplement**

The epidemiological model implemented by the *FluEvidenceSynthesis* package (20) is as follows:

$$\frac{dS_{i}}{dt}=-\lambda_{i}S_{i}$$

$$\frac{dE_{i}^{1}}{dt}=\lambda_{i}S_{i}-\gamma_{1}E_{i}^{1}$$

$$\frac{dE_{i}^{2}}{dt}=\gamma_{1}{(E}_{i}^{1}-E_{i}^{2})$$

$$\frac{dI_{i}^{1}}{dt}=\gamma_{1}E_{i}^{2}-\gamma_{2}I_{i}^{1}$$

$$\frac{dI_{1}^{2}}{dt}=\gamma_{2}\left( I_{i}^{1}-I_{i}^{2} \right)$$

$$\frac{dR_{i}}{dt}=\gamma_{2}I_{i}^{2}$$

where $S_{i}$ is the number of susceptibles in age group i, $E_{i}^{1}$ and $E_{i}^{2}$ are two compartments with exposed but not yet infectious individuals in age group i, $I_{i}^{1}$ and $I_{1}^{2}$ are infectious individuals and $R_{i}$ are immune individuals in age group i. The two compartments for the exposed and infectious states result in a more realistic gamma distributed average time for these states than is given by the usual exponential distribution. The overall rate of loss of latency and infectiousness are given by $\gamma_{1}$/2 and $\gamma_{2}$/2 respectively.

The age-group specific force of infection, $\lambda_{i}$ , is given by

$$\lambda_{i}=\sigma_{i}\sum_{j=1}^{6} \beta_{ij}(I_{j}^{1}+I_{j}^{2})$$

where $\sigma_{i}$ is the susceptibility of age group i and $\beta_{ij}$ is the effective contact rate between individuals in age group i and age group j. $\beta_{ij}$ is derived by multiplying the transmissivity by the probability of a contact between individuals in the two age groups.

A harmonic seasonal variation in transmissivity was implemented as an amendment to the *FluEvidenceSynthesis* model

$$\tau\left( t \right)=\tau_{B}+\tau_{V}cos(2\pi\left( t-t_{max} \right))$$

where $\tau\left( t \right)$is the time varying transmissivity, $\tau_{B}$ is the baseline transmissivity, $\tau_{V}$ is the seasonal amplitude of transmissivity variation and $t_{max}$ is the phase i.e. the day of the year where transmissivity is at a maximum.

The impact of vaccination is implemented by further separating each of the epidemiological compartments (SEEIR) into vaccinated and unvaccinated groups. The full equations for both vaccinated and unvaccinated groups can be found in Baguelin et al (16).

**Data supplement:**

**Estimates of health-seeking behaviours for influenza**

For a disease like influenza, depending on the symptoms (which can vary from mild to severe), a proportion of the infected population will visit their GP or present to the ED. Of those who present to the ED, some will be admitted to hospital.

**Estimates used for calculating primary care (GP) presentation rate, emergency department (ED) presentation rate and ICU admission rate for influenza by age-group**

|  | <5 years | 5-11 years | 12-17 years | 18-44 years | 46-64 years | ≥65 years + |
| --- | --- | --- | --- | --- | --- | --- |
| **Primary care presentation rate^a^** | 67% | 52% | 51% | 37% | 43% | 56% |
| **ED admission rate: proportion of ED presentations who have hospital admission^b^** | 18.9% | 13.3% | 13.3% | 23.6% | 35.0% | 58.0% |
| **ICU admission rate: proportion of hospitalisations admitted to ICU^c^** | 12.2% | 10.5% | 18.3% | 12.1% | 15.7% | 8.7% |

**Sources**

^a^ Matthew Biggerstaff, Michael Jhung, Laurie Kamimoto, Lina Balluz, Lyn Finelli. Self-Reported Influenza-Like Illness and Receipt of Influenza Antiviral Drugs During the 2009 Pandemic, United States, 2009–2010, American Journal of Public Health 102, no. 10 (October 1, 2012): pp. e21-e26. <https://ajph.aphapublications.org/doi/epdf/10.2105/AJPH.2012.300651>

^b^ Australian Institute of Health and Welfare 2018. Emergency department care 2017–18: Australian hospital statistics. Health services series no. 89. Cat. no. HSE 216. Canberra: AIHW. Available from: <https://www.aihw.gov.au/getmedia/9ca4c770-3c3b-42fe-b071-3d758711c23a/aihw-hse-216.pdf.aspx>

^c^ Data (2011-2019) from Influenza Complications Alert Network (FluCAN). See Allen Cheng, Dominic Dwyer, Mark Holmes, Louis Irving, Graham Simpson, Sanjaya Senenayake et al Influenza epidemiology in patients admitted to sentinel Australian hospitals in 2019: the Influenza Compliations Alert Network (FluCAN)., Commun Dis Intell (2018). <https://www1.health.gov.au/internet/main/publishing.nsf/Content/2A15CD097063EF40CA2587CE008354F1/$File/influenza_epidemiology_in_patients_admitted_to_sentinel_australian_hospitals_in_2019_the_influenza_complications_alert_network_flucan.pdf>

**Length of stay by age-group**

| **Age-group** | **Mean Hospital length of stay (ICU + non-ICU) (days)** | **Mean ICU Length of stay** |
| --- | --- | --- |
| <5 years | 4.47 days | 5.31 days |
| 5-11 years | 4.30 days | 5.95 days |
| 12-17 years | 6.03 days | 7.43 days |
| 18-44 years | 5.17 days | 5.59 days |
| 45-64 years | 7.19 days | 7.53 days |
| ≥65 years | 7.63 days | 6.61 days |

**Source**

^c^ Data (2011-2019) from Influenza Complications Alert Network (FluCAN). See Allen Cheng, Dominic Dwyer, Mark Holmes, Louis Irving, Graham Simpson, Sanjaya Senenayake et al Influenza epidemiology in patients admitted to sentinel Australian hospitals in 2019: the Influenza Complications Alert Network (FluCAN)., Commun Dis Intell (2018). <https://www1.health.gov.au/internet/main/publishing.nsf/Content/2A15CD097063EF40CA2587CE008354F1/$File/influenza_epidemiology_in_patients_admitted_to_sentinel_australian_hospitals_in_2019_the_influenza_complications_alert_network_flucan.pdf>

**Estimates of costs**

|  | **Costs** |
| --- | --- |
| **Hospital costs** | **Emergency department ^d^**   - Average cost per admitted presentation was A$1,464 - Average cost per non-admitted presentation was A$675   **Hospital admissions ^e,f^**  A$2665.45 per non-ICU bed-day (A$5864 for a mean length of stay of 2.2 non-ICU bed-days) ^e^  A$4375 per ICU bed-day ^f^ |
| **Primary Health Costs** | Cost of the GP appointment: A$38.75 **^g^** |
| **Vaccine delivery costs** | Cost of the physical vaccine: A$10  Cost of the GP appointment where it is administered: A$38.75 **^g^**  Total costs: A$48.75 |

**Source**

^d^ NHCDC, round 23 (2018-2019), Table 12

<https://www.ihpa.gov.au/publications/national-hospital-cost-data-collection-report-public-sector-round-23-financial-year>

^e^ NHCDC, round 23 (2018-2019), Table 8

<https://www.ihpa.gov.au/publications/national-hospital-cost-data-collection-report-public-sector-round-23-financial-year>

^f^ Hicks P, Huckson S, Fenney E, Leggett I, Pilcher D, Litton E^.^ [The financial cost of intensive care in Australia: a multicentre registry study.](https://pubmed.ncbi.nlm.nih.gov/31420872/) Med J Aust. 2019 Oct;211(7):324-325. doi: 10.5694/mja2.50309. Epub 2019 Aug 16.

^g^ MBS – item 23 <http://www9.health.gov.au/mbs/fullDisplay.cfm?type=item&qt=ItemID&q=23#assocNotes>
